# Development of a novel bioactive glass for air-abrasion to selectively remove orthodontic adhesives

**DOI:** 10.1007/s00784-017-2279-8

**Published:** 2017-11-28

**Authors:** Ayam A. Taha, Robert G. Hill, Padhraig S. Fleming, Mangala P. Patel

**Affiliations:** 1 Dental Physical Sciences, Barts and the London School of Medicine and Dentistry, Institute of Dentistry, Queen Mary University of London, Mile End Road, London, E1 4NS UK; 2 Department of Paedodontic, Orthodontic and Preventive Dentistry, College of Dentistry, Al-Mustansiriya University, Baghdad, Iraq; 3 Department of Orthodontics, Barts and the London School of Medicine and Dentistry, Institute of Dentistry, Queen Mary University of London, Turner St, London, E1 2AD UK

**Keywords:** Orthodontic adhesive removal, Bioactive glass, Air-abrasion

## Abstract

**Objectives:**

To develop a novel, bioactive glass for removing residual orthodontic adhesive via air-abrasion, following bracket debonding, and to evaluate its effectiveness against a proprietary bioactive glass 45S5(Sylc™)-air-abrasion, and a slow-speed tungsten carbide (TC) bur.

**Materials and methods:**

Three glasses were prepared and their bioactivity was proved. One novel glass (QMAT3) was selected due to its appropriate hardness, lower than that of enamel/45S5(Sylc™). Sixty extracted human premolars were randomly assigned to adhesive removal using: (a) QMAT3-air-abrasion, (b) 45S5(Sylc™)-air-abrasion, and (c) TC bur, which were further subdivided (*n* = 10) based on the adhesive used (Transbond XT™ or Fuji Ortho LC™). Enamel roughness was assessed using scanning electron microscopy (SEM) and non-contact profilometry before bracket bonding, after removing residual adhesive following bracket debonding and after polishing.

**Results:**

QMAT3 formed apatite faster (6 h) than 45S5(Sylc™) (24 h) in Tris solution. QMAT3-air-abrasion gave the lowest enamel roughness (Ra) after removing the adhesives. SEM images showed a pitted, roughened enamel surface in the TC bur group and to a lesser extent with 45S5(Sylc™), while a virtually smooth surface without any damage was observed in the QMAT3-air-abrasion group. The time taken for adhesive removal with QMAT3 was comparable to 45S5(Sylc™) but was twice as long with the TC bur.

**Conclusions:**

QMAT3-air-abrasion is a promising technique for selective removal of adhesives without inducing tangible enamel damage.

**Clinical relevance:**

A novel bioactive glass has been developed as an alternative to the use of TC burs for orthodontic adhesive removal.

## Introduction

Several factors may predispose to or directly induce enamel damage during, or after, fixed orthodontic treatment. The post clean-up procedure after removal of attachments is regarded as the most significant cause of enamel damage [1, 2]. Therefore, various methods have been proposed for clean-up of residual orthodontic adhesives from the enamel surface, such as: hand instruments, stones (Arkansas stone, green stone), wheels and discs, scalers, dental burs (typically tungsten carbide burs), lasers and pumice or zirconium paste [3]. Currently, no technique has proven capable of complete and efficient removal of residual adhesives, without inducing even a minor amount of enamel damage [4]. These surface changes reduce the resistance of enamel to bacterial/organic acid attacks therefore increasing its susceptibility to demineralization and dental caries.

The critical threshold value of enamel surface roughness for bacterial adhesion has been established at 0.2 μm by Bollen et al. [5]. A number of studies have shown that conventional methods of adhesive removal, including scalers and dental burs, may lead to visible surface roughness with gouges ranging from 10–20 μm deep, and loss of up to 100 μm thickness of enamel [6]. Thus, maintaining the integrity of the enamel surface during the removal of residual adhesive is a key consideration during the removal of orthodontic appliances.

In recent years, air-abrasion has shown promise as a method for removing residual adhesives [7]. Banerjee and his co-workers, in an in vitro air-abrasion study, reported that the bioactive glass powder 45S5 produced less enamel damage compared with alumina air-abrasion and tungsten carbide burs [7]. However, there still remains a need to improve the properties of bioactive glasses to facilitate the safe removal of residual adhesives, with minimal/no enamel damage, following bracket debonding.

The aims of the present study were therefore:

i. To develop a novel fluoride-containing bioactive glass with a hardness lower than that of the bioactive glass 45S5(Sylc™) and that of sound enamel surface, and
ii. To study the effectiveness of the novel glass in the removal of residual orthodontic adhesives from the enamel surface, when propelled via an air-abrasion hand-piece, in comparison with a TC bur and 45S5(Sylc™)-air-abrasion.

## Material and methods

### Glass design and synthesis

A series of three novel glasses incorporating SiO_2_− P_2_O_5_−CaO−Na_2_O−CaF_2_, based on the molar composition of a commercially-used bioactive glass 45S5(Sylc™; Denfotex Research Ltd., London, UK) were synthesized using a melt quench route (Table 1). The Na_2_O content was systematically increased (in exchange for CaO) up to 30 mol%. High-phosphate content (6.1 mol% P_2_O_5_) was also used. In addition, a constant ratio of calcium fluoride (3 mol% CaF_2_) was added. The network connectivity value was kept constant (2.08) for all the experimental glasses. Each glass (batch size 200 g) was prepared by melting SiO_2_ (analytical grade; Prince Minerals Ltd., Stoke-on-Trent, UK), Na_2_O, CaO, P_2_O_5_, and CaF_2_ (Sigma-Aldrich, Gillingham, UK) in a platinum-rhodium crucible, in an electrical furnace (EHF 17/3, Lenton, UK) for 60 min between 1420 to 1450 °C (Table 1). The resulting molten glass was rapidly quenched in deionized water (DW) to obtain glass frits, which were collected into a sieve and kept in a vacuum oven (Harvard LTE, UK) to dry at 80 °C overnight.

**Table 1:** Nominal glass composition (in Mol %) and melting temperature (Tm)

Glasses	Mol %					Tm
	SiO_2_	Na_2_O	CaO	P_2_O_5_	CaF_2_	
45S5(Sylc™)	46.1	24.4	26.9	2.6		1450
QMAT1	37	20	33.9	6.1	3	1440
QMAT2	37	25	28.9	6.1	3	1430
QMAT3	37	30	23.9	6.1	3	1420

After drying, 100 g of each glass frit was ground using a vibratory mill (Gy-Ro mill, Glen Creston, London, UK) for 1 min to form glass powders, which were then sieved for 10 min (38 and 90 μm mesh analytical sieves; Endecotts, Ltd., London, UK), to obtain glass particle size fractions between 38 and 90 μm. Each glass powder was stored in dry re-sealable plastic bags until further use. Glass particles of < 38 μm size were used for bioactivity tests to assess apatite formation (in Tris buffer solution), whereas glass particles between 38 and 90 μm were used for propulsion via the air-abrasion hand-piece. This range of particle size allowed escape of the glass powder through the hand-piece nozzle tip without agglomeration. Bioactive glass 45S5(Sylc™) was used as a reference with a particle size less than 38 μm for bioactivity tests and between 38 and 90 μm for air-abrasion studies.

### Glass characterization

X-ray diffraction (XRD; X’Pert PRO MPD, PANalytical, Cambridge, UK; 40 kV/40 mA, Cu Kα, data collected at room temperature; results not shown) was used to determine the amorphous state of the glass. The glass transition temperature (Tg) was determined for each glass composition using Differential Scanning Calorimetry (DSC; Stanton Redcroft DSC1500, Rheometric Scientific, Epsom, UK). 50 mg (± 0.1 mg) of glass powder (< 38 μm) was placed in a DSC platinum crucible and run against alumina powder (analytical grade) as a reference, at a heating rate 20 °C per minute in flowing nitrogen gas (flow rate of 60 ml/min), from 25 to 1000 °C.

### Glass Vickers hardness measurements

For hardness measurements, a glass rod (20 mm in diameter) was prepared from each glass batch, by re-melting approximately 100 g of glass frit, pouring into a graphite mould, and annealed for 1 h in a preheated furnace, at the Tg determined in 2.2. Thereafter, the casting glass was slowly cooled to room temperature overnight in the furnace, which was switched off. The rod from each glass batch was sectioned into approximately 1-mm thick discs using a diamond cutter machine (Accutom-5, Struers A/S, Ballerup, Denmark). These discs were subsequently polished with silicon carbide paper (P1000 in roughness) wet with acetone (instead of water), to avoid the glass reacting with water during polishing. The hardness of the discs was measured using a Vickers diamond pyramid indenter (Zwick/Roell, ZHU 187.5) with an applied load of 29.4 N for 10 s. The Vickers hardness number (VHN) of each glass composition was taken 10 times. The VHN values (displayed on the LCD) were averaged and presented as mean ± standard deviation (SD). QMAT3 experimental glass was selected for air-abrasion tests on the basis of its lower hardness compared to that of enamel and bioactive glass 45S5(Sylc™).

### Glass particle size and shape

Particle size analysis was performed on glass powders using a Malvern/E Mastersizer (Malvern instruments, UK), since air-abrasion technique utilizes kinetic energy, which depends on the mass (size) and the velocity of the propelled glass powder particles. Approximately 30 mg of the glass powder was dispersed in DW until the ideal laser absorbance level was achieved. Two measurements were recorded and the average of these measurements was taken to produce a more reliable value. For glass particle shape, a scanning electron microscope (SEM-FEI Inspect F, Oxford instruments, UK) with an accelerating voltage of 20 kV and a working distance of 10 mm was used after mounting the glass particles on stubs, and sputter-coated with gold using an automatic sputter coater (SC7620, Quorum Technologies, UK).

### Apatite formation in Tris buffer

Tris buffer solution was prepared (15.09 g Tris (hydroxylmethyl) aminomethane powder, 800 ml of DW and 44.2 ml of 1 M hydrochloric acid—Sigma Aldrich), and shaken (orbital shaker; IKA® KS 4000i control, Germany) at 37 °C overnight. The pH of the solution was measured using a pH meter (Oakton Instruments), and adjusted to ~ 7.25–7.4 (with 1 M hydrochloric acid); the volume of Tris buffer solution was increased to 2 litres by adding DW and stored in an incubator at 37 °C. In order to observe apatite formation of each glass powder, the latter was dispersed (75 mg; with a particle size < 38 μm) into 50 ml of Tris buffer solution in a polyethylene bottle (150 ml). These bottles were then kept in a shaking incubator at 37 °C, with a rotation rate of 60 rpm, for the following time intervals: 1, 3, 6, 9, and 24 h.

After the designated time intervals, the glass powders immersed in Tris buffer solution were filtered (through filter paper; Fisher brand® qualitative filter paper). The glass powder within the filter paper was then placed in an oven at 37 °C for 24 h to be dried. The dried powders were subsequently analysed using Fourier transform infrared spectroscopy (FTIR; ~ 5 mg of each glass powder; < 38 μm) and X-ray diffraction (XRD; 1 to 20 mg of each glass powder, with a particle size < 38 μm). The FTIR (Spectrum GX, Perkin-Elmer, Waltham, USA) data was collected between from 500 to 1800 cm^−1^_,_ absorbance mode. The XRD (X’Pert PRO MPD, PANalytical, Cambridge, UK; 40 kV/40 mA, Cu Kα) data was collected at room temperature in the 2*θ* range of 10° to 70°.

### Tooth sample preparation

Sixty human premolars, extracted for orthodontic purposes, were used (with approval from Queen Mary Research Ethics Committee QMREC 2011/99). These teeth were selected on the basis of visual observation using an optical stereo-microscope at ×4.5 magnification (VWR International Microscope). The inclusion criteria were no carious lesions, cracks or any other defects on the buccal surfaces. The selected teeth were cleaned and stored in DW in a refrigerator at 4 °C until required. Prior to the start of the experiment, these teeth were washed with DW, air-dried and embedded into plastic moulds filled with cold cure acrylic resin (Orthocryl™, UK), leaving the buccal surfaces exposed. The buccal surface of each tooth sample was polished with non-fluoridated pumice paste (20 s), rinsed with water and air-dried. Thereafter, a polyvinyl chloride tape was placed on the buccal surface of each tooth sample, excluding a 4 × 4 mm window at the centre for bonding of orthodontic brackets to the exposed enamel. The covered area was used as a reference for later visual comparison between the treated and untreated surfaces. Finally, these prepared teeth samples were stored in an incubator at 37 °C until the brackets were bonded.

### Orthodontic adhesive removal

Following the manufacturer’s instructions, two light-cured orthodontic adhesive systems: resin composite; Transbond XT™ (3 M Unitek, Monrovia, CA, USA), and resin modified glass ionomer cement; Fuji Ortho LC™ (GC corporation, Tokyo, Japan) were used to bond 60 premolar metal brackets (MiniSprint®, Forestadent, Pforzheim, Germany) to the prepared teeth samples (30 premolar teeth per each orthodontic adhesive system group). Enamel etching with 37% phosphoric acid was undertaken for 30 s prior to application of Transbond XT™. When the brackets were placed, each bracket was subjected to a 300 g compressive force using a force gauge (Correx Co, Berne, Switzerland) for 5 s, to ensure a uniform thickness of the adhesive [8]. After bonding, the teeth with the attached brackets were stored in DW for 1 week at 37 °C. Thereafter, the plastic moulds (with the extracted teeth mounted) were held by a special holding device (Instron® machine, UK), to remove the attached brackets from the buccal surfaces using a debonding plier (Ixion™, DB Orthodontics) by one operator. Three different clean-up methods (slow-speed tungsten carbide bur (TC), 45S5(Sylc™)-air-abrasion, and the selected experimental glass (QMAT3)-air-abrasion were used for removal of the residual of the aforementioned two orthodontic adhesive systems following bracket debonding. The teeth samples of each orthodontic adhesive system group were randomly assigned to three groups (ten teeth for each post clean-up method). Both the commercially available bioactive glass 45S5(Sylc™) and QMAT3 glass were propelled via an air-abrasion hand-piece (BA Ultimate air polisher) connected to a dental chair unit. The operating parameters were air-pressure 60 psi, nozzle angle 75° and nozzle tip-enamel surface distance of 5 mm. Complete removal of the adhesive remnants was assessed by visual inspection under a dental operating light and later verified by an optical stereo-microscope at ×4.5 magnification (VWR International Microscope). A non-contact white light profilometery (Proscan®2000, Scantron, Taunton, UK) was used to measure the enamel surface roughness for all prepared teeth samples before bracket bonding, after post clean-up method, and after polishing (using rubber cup and non-fluoridated pumice for 20 s). In addition, scanning electron microscopy (SEM-FEI Inspect F, Oxford instruments, UK) was used to examine the enamel surface damage following the aforementioned post clean-up methods, and the time required to remove adhesive remnants was also assessed.

### Statistical analysis

Statistical analysis was performed with the SPSS software package (version 24; SPSS Inc., New York, NY, USA). Two-way analysis of variance (ANOVA) and Tukey’s HSD post-hoc test were used to test significant differences in enamel surface roughness under three different conditions (before bracket bonding, after post clean-up methods following bracket debonding, and after polishing). The level of significance was pre-specified at alpha = 0.05.

## Results

### Glass characterization and Vickers hardness measurements

The glass transition temperature (Tg) of both 45S5(Sylc™) and experimental glasses are shown in Table 2. The Tg was 530 °C for 45S5(Sylc™) with sodium and phosphate contents of 24.4 and 2.6 mol%, respectively. The Tg of QMAT3 reduced to 355 °C as the sodium and phosphate increased to 30 and 6.1 mol%, respectively. This was also associated with the addition of a constant ratio of fluoride (3 mol%). Furthermore, the Vickers hardness number (VHN) decreased dramatically (Table 2) from 472.8 ± 2.28VHN (~ 4.63GPa) for 45S5(Sylc™) to 350.4 ± 1.14VHN (~ 3.43GPa) for QMAT3. The experimentally determined VHN were converted to GPa units using the equation: GPa = VHN × 0.009807.

**Table 2:** Glass transition temperature (Tg), and Vickers hardness number (VHN) of 45S5(Sylc™) and experimental glasses

Bioactive glasses	Tg (°C)	Vickers hardness (VHN) mean ± SD	Hardness (GPa)
45S5(Sylc™)	530	472.8 ± 2.28	4.63
QMAT1	524	458.6 ± 2.50	4.49
QMAT2	450	433.6 ± 1.94	4.25
QMAT3	355	350.4 ± 1.14	3.43

### Glass particle size and shape

The particle size distribution (in micrometres; μm) of 45S5(Sylc™) glass and experimental glasses are given in Table 3, where D10 represents 10% of the glass particle size, indicating the fine particles within the distribution D50 represents 50% of the glass particle size, giving a measure of the mean particle size within the distribution and D 90 represents 90% of the glass particle size, reflecting larger particle sizes.

**Table 3:** Particle size distribution of 45S5(Sylc™) and experimental glasses

Bioactive glasses	Particle size (μm)		
	D10	D50	D90
45S5(Sylc™)	34.1	63.5	76.8
QMAT1	38.6	65.5	77.2
QMAT2	38.9	65.5	77.2
QMAT3	33.8	62.7	76.7

**Fig. 1:**
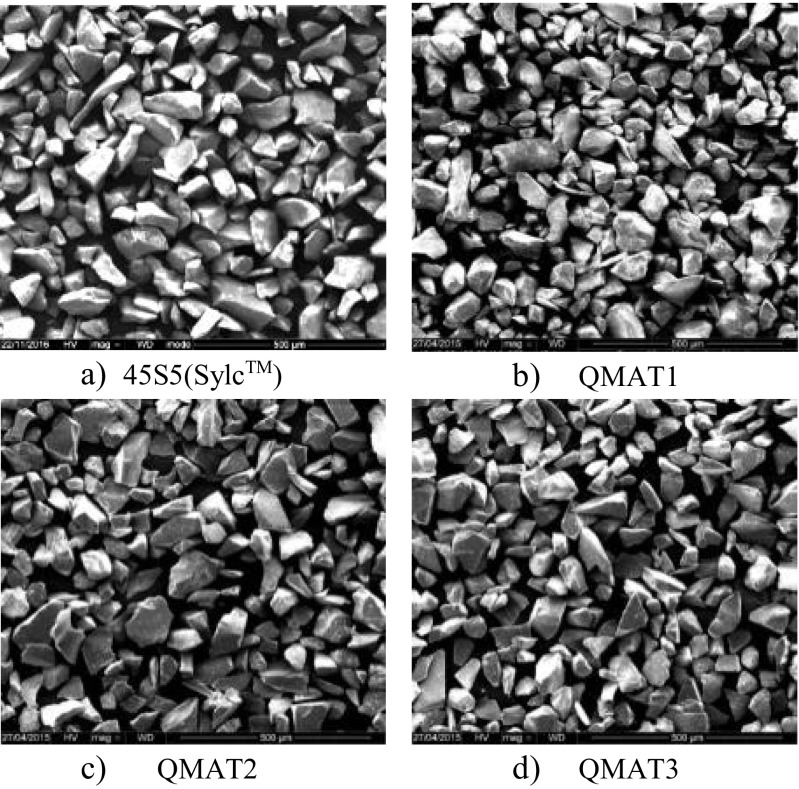
SEM images of (**a**) 45S5(Sylc™), (**b**) QMAT1, (**c**) QMAT2, and (**d**) QMAT3 at ×250 magnification

SEM images of 45S5(Sylc™) and experimental glasses are presented in Fig.1. All glasses show similar morphology, represented as sharp, angular irregular particles of variable sizes (in micrometres).

### Apatite formation in Tris buffer

**Fig. 2:**
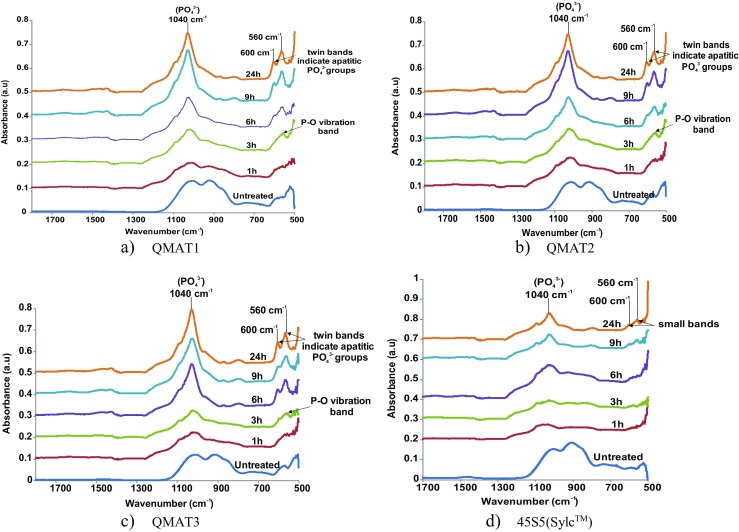
FTIR spectra for (**a**) QMAT1, (**b**) QMAT2, (**c**) QMAT3 and (**d**) 45S5(Sylc™) after immersion in Tris buffer solution

After immersion in Tris buffer solution for 1, 3, 6, 9 and 24 h, the FTIR spectra for all experimental glasses showed dramatic changes compared to their respective untreated (not immersed) versions. The latter were characterized by the presence of two main bands at 910 and 1040 cm^−1^, indicating non-bridging oxygen (Si^+^-O^-^- M^+^, where M^+^ is an alkali metal modifier element) and vibrational stretching of Si-O-Si, respectively [9, 10]. After immersion, the non-bridging oxygen (Si^+^-O-M^+^) band at 910 cm^−1^ disappeared, and a single P–O vibration band at 560 cm^−1^ appeared after 3 h, which is an indicator for the presence of apatite precursors [9]. At 6 h, the latter band split into prominent twin bands at 560 and 600 cm^−1^, which became well-defined with longer immersion times. These twin bands indicate the presence of apatitic PO_4_^3−^groups, the main characteristic feature of apatite, including hydroxyapatite, fluorapatite and carbonated hydroxyapatite [11, 12]. The formation of apatite was confirmed by the presence of a sharp phosphate band (PO_4_)^3−^ at 1040 cm^−1^ after 6, 9 and 24 h of immersion [9]. Conversely, 45S5(Sylc™) did not show any bands at 560 and 600 cm^−1^, with the absence of the sharp phosphate band at 1040 cm^−1^ at 6 h. Apatite formation features of 45S5(Sylc™) appeared at 24 h but these were still less prominent compared to those obtained for all experimental glasses (Fig.2).

**Fig. 3:**
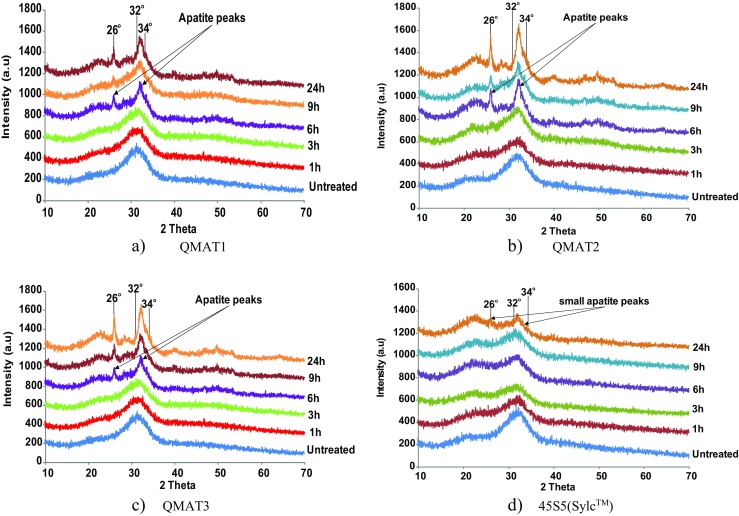
XRD data for (**a**) QMAT1, (**b**) QMAT2, (**c**) QMAT3 and (**d**) 45S5(Sylc™) after immersion in Tris buffer solution

With regard to XRD patterns, all the experimental glasses showed a small peak at 26° and a broad peak from 32° to 34° 2*θ* after 6 h of immersion in Tris buffer solution, superimposing the amorphous broad peak of untreated glass and those that were immersed for 1 and 3 h (Fig.3). These two peaks, indicating the presence of apatite, became more pronounced as the immersion time increased. However, all these peaks were absent in 45S5(Sylc™) until 24 h, when much smaller peaks indicative of apatite appeared [13].

### Orthodontic adhesive removal

The profilometry analysis was carried out on three experimental groups (TC group, 45S5(Sylc™)-air-abrasion, and QMAT3-air-abrasion) for each orthodontic adhesive. The means and standard errors (SE) of the average enamel surface roughness (Ra) in micrometers, under three different conditions, are presented in Table 4. No statistically significant differences were observed in the enamel roughness measurements among the six experimental study groups before bracket bonding (baseline measurements). For Transbond XT™ resin groups, the enamel roughness significantly increased after post clean-up with TC bur (2.93 ± 0.06 μm), and 45S5(Sylc™) (1.89 ± 0.04 μm) compared with their corresponding baseline measurements (*p* < 0.001), while QMAT3-air-abrasion group did not exhibit any significant difference in enamel roughness (*p* = 0.927). In addition, enamel roughness values after polishing were significantly higher for both the TC and 45S5(Sylc™)-air-abrasion groups (2.73 ± 0.77 μm and 1.81 ± 0.05 μm, respectively), than their corresponding baseline measurements (*p* < 0.001). However, no significant difference was shown following clean-up with QMAT3-air-abrasion and subsequent polishing and baseline values (*p* = 0.983). Furthermore, enamel roughness measurements did not appear to be affected by polishing subsequent to clean-up in each group (*p* = 0.104 to 1.000) compared to those after clean-up.

**Table 4:** Enamel surface roughness (Ra) in micrometers (mean ± SE) for each experimental group under three different conditions

Group (*n* = 10)	Experimental group based on: orthodontic adhesive + post clean-up method used	Before bracket bonding (baseline)	After post clean-up method	After polishing
1	Transbond XT™ + TC	0.49 ± 0.27	2.93 ± 0.06	2.73 ± 0.77
2	Transbond XTT^M^ + 45S5(Sylc™)-air-abrasion	0.51 ± 0.03	1.89 ± 0.04	1.81 ± 0.05
3	Transbond XT™ + QMAT3-air-abrasion	0.49 ± 0.04	0.58 ± 0.02	0.56 ± 0.03
4	Fuji Ortho LC™ + TC	0.54 ± 0.02	2.57 ± 0.05	2.63 ± 0.06
5	Fuji Ortho LC™ + 45S5(Sylc™)-air-abrasion	0.46 ± 0.04	1.59 ± 0.02	1.74 ± 0.04
6	Fuji Ortho LC™ + QMAT3-air-abrasion	0.36 ± 0.02	0.51 ± 0.04	0.45 ± 0.01

With Fuji Ortho™ resin modified glass ionomer cement, similar patterns were observed for enamel roughness measurements. They were significantly higher after post clean-up in the TC (2.57 ± 0.05 μm) and 45S5(Sylc™)-air-abrasion groups (1.59 ± 0.02 μm) compared to baseline measurements (*p* < 0.001), but the QMAT3-air-abrasion group (0.51 ± 0.04 μm) did not show any significant difference with their corresponding baseline measurements. In addition, significantly higher enamel roughness values were shown in TC and 45S5(Sylc™)-air-abrasion groups (2.63 ± 0.06 μm and 1.74 ± 0.04 μm, respectively) after polishing than their corresponding baseline measurements (*p* < 0.001), but no significant difference was shown between those of QMAT-air-abrasion and their corresponding baseline measurements (*p* = 1.000).

The time required to remove the orthodontic adhesives was recorded for three post clean-up methods: TC bur, Sylc™-air-abrasion, and QMAT3-air-abrasion (Table 5). Differences between QMAT3 glass (42.51 ± 1.11 s) and Sylc™ (40.72 ± 0.92 s) were not statistically significant (*p* = 0.913) in the Transbond XT™ groups. However, both took longer (*p* < 0.001) to remove excess Transbond XT™ resin compared to the TC bur (23.2 ± 1.58 s). A similar pattern was observed in the Fuji Ortho LC™ groups with no significant differences (*p* = 0.893) found between the time required to remove Fuji Ortho LC™ by both QMAT3 and Sylc™ glasses, while both glasses took significantly longer than the TC bur (*p* < 0.001).

**Table 5:** Means ± SE of the time (seconds) required to remove two residual orthodontic adhesives after bracket debonding

Group (*n* = 10)	Experimental study group based on: orthodontic adhesive + post clean-up method used	Time (Sec.)
1	Transbond XT™ + TC	23.20 ± 4.99
2	Transbond XT™ + 45S5(Sylc™)-air-abrasion	40.71 ± 2.89
3	Transbond XT™ + QMAT3-air-abrasion	42.51 ± 3.51
4	Fuji Ortho LC™ + TC	22.90 ± 4.41
5	Fuji Ortho LC™ + 45S5(Sylc™)-air-abrasion	38.42 ± 4.29
6	Fuji Ortho LC™ + QMAT3-air-abrasion	40.32 ± 3.36

**Fig. 4:**
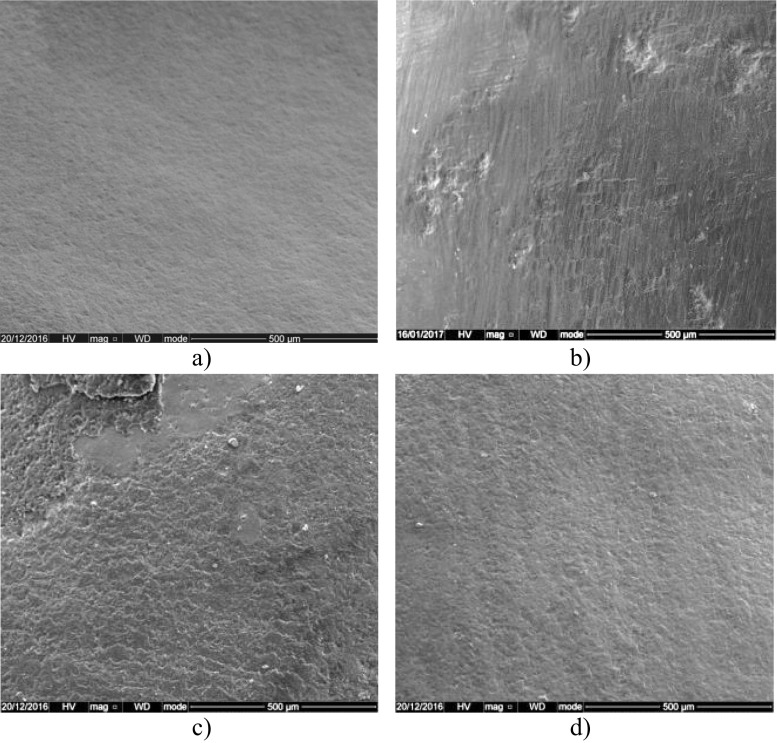
Representative SEM images (×250 magnification) of the enamel surface. (**a**) Before bracket bonding. (**b**) After clean-up using the TC bur. (**c**) After clean-up using 45S5(Sylc™)-air-abrasion. (**d**) After clean-up using QMAT3-air-abrasion

Representative SEM images (at ×250 magnification) are shown in Fig.4 prior to bracket bonding and after the three post clean-up methods (TC bur, 45S5(Sylc™)-air-abrasion, and QMAT3-air-abrasion). The sound enamel surface appeared smooth before bracket bonding (Fig.4a), whilst it became roughened and pitted surface after the use of a slow-speed TC bur (Fig.4b). In addition, the enamel surface, following 45S5(Sylc™)-air-abrasion is seen to have microscopic roughness in some areas (Fig.4c), while a uniformly smooth surface was obtained after using QMAT3-air-abrasion (Fig.4d).

## Discussion

### Glass characteristics

Bioactive glass, 45S5, has been used in a number of commercial products such as NovaBone™ (orthopaedic application), Perioglass™ (periodontal application), Sylc™ and NovaMin™ (dental application) [14]. Furthermore, preliminary in vitro research has been undertaken on its use via propulsion using air-abrasion, in order to cut sound and carious enamel and dentine [15, 16], remove residual orthodontic adhesive following bracket debonding from enamel surfaces [7] and potentially re-mineralize white spot lesions [17]. However, this glass has higher hardness than that of sound enamel (~ 3.5GPa) [18] with reported values varying between 4.5GPa [19] and 5.75GPa [20], thus risking roughening of the enamel surface. Therefore, it is desirable to develop glasses which have similar or lower hardness than that of enamel.

It has been reported that increasing Na_2_O content (in exchange for CaO), across a series of bioactive glasses, with a constant network connectivity value close to two, resulted in a linear decrease in Tg and density of the glass [21, 22]. This was attributed to the substitution of CaO for Na_2_O producing a more disrupted silicate glass network, since one Ca^2+^ was replaced by two Na^+^ ions. This resulted in the loss of the ionic bridges that Ca^2+^ ions provided between two adjacent non-bridging oxygens, contributing to a decrease in the packing density of the glass. Therefore, less rigid glasses were formed with a lower glass transition temperature (Tg) to transform them from a molten liquid to a glassy state.

Additionally, it has also been reported that a reduction in silica content, characteristic of all experimental glasses tested compared to the proprietary bioactive glass 45S5(Sylc™), resulted in a decrease in Tg [23]. In the latter study, a reduction in silica content was accompanied by an increase in phosphate content. Similarly, a range of studies [13, 14, 24] have reported a decrease in Tg related to a reduction in silica content accompanied by an increase in phosphate content and the addition of fluoride. Moreover, the addition of fluoride to bioactive glasses has also been reported to lower the Tg as well as to promote fluorapatite formation, which is more stable and resistant to acid attack, compared with hydroxyapatite [25, 26].

Fluoride, in the form of CaF_2_, was incorporated in the composition of all experimental glasses in this study. The amount of CaF_2_ was kept constant (3 mol%) to enhance fluorapatite formation and prevent fluorite development (as a result of excessive fluoride; ≥ 5 mol%) [13, 26, 27]. The formation of fluorite indicates crystallization of the glass, resulting in inhibition of its bioactive properties; the presence of crystalline phases gives rise to increased resistance to ion exchange reactions between the glass surface and physiological solution, which in turn affect apatite formation. Furthermore, the phosphate content in this study was also increased from 2.6 mol% in the reference glass 45S5(Sylc™) to 6.1 mol% in all experimental glasses. This formulation was informed by two recent studies [13, 28] indicating that increasing the phosphate content to approximately 6 mol% in fluoride-containing glasses resulted in an increase in the degradation of the glass, leading to a rapid release of calcium (Ca^2+^) and orthophosphate (PO4^3−^) ions to the surrounding solution, thus forming fluorapatite within 6 h after immersion in Tris buffer solution. The results of this study regarding apatite formation are consistent with these findings.

Interestingly, it was also evident that increasing the Na_2_O content in the present study, resulted in a decrease in hardness values for all experimental glasses to (3.43GPa for QMAT3) compared with the reference 45S5(Sylc™; 4.63 GP). Hence, QMAT3 conserved the enamel surface while removing the residual adhesives, without inducing undesirable surface enamel changes. It should be noted that the available information on the hardness values of bioactive glasses is limited due to the relatively small number of studies investigating this property. Reported values vary significantly from one study to another; for example, the hardness for bioactive glass, 45S5 reported by Lopez-Esteban et al. [20] and Farooq et al. [28] were 5.75GPa and 5.84GPa, respectively. This variation might be due to the differences in the methodology used to prepare these glasses, and the indenter load used to measure the hardness, although all studies used a Vickers’s instrument. Moreover, in the current study the indenter load was 2.9 kg while the indenter loads used by Lopez-Esteban et al. [20] and Farooq et al. [28] were 0.5–1.2 and 0.01 kg, respectively.

Ninety per cent of each experimental glass (QMAT1, QMAT2 and QMAT3) had a particle size of 77.2, 77.2 and 76.7 μm, respectively. These values were close to those for 45S5(Sylc™; 76.8 μm). The larger mass (particle size) leads to higher kinetic energy since the latter relates to the mass (particle size) and velocity. In addition, the presence of large particles and the absence of fine particles prevented agglomeration of the glass powder, since fine particles can result in clumping and stagnation of the glass powder within the nozzle tip of the air-abrasion hand-pieces, thus potentially hindering the air-abrasion process.

With regard to the morphology of the commercially-available 45S5(Sylc™) glass and experimental glasses, the SEM images revealed a very similar appearance characterized by sharp, angular and irregular particles, thus aiding in removing the residual of orthodontic adhesives.

The FTIR spectra and XRD patterns of 45S5(Sylc™) and all experimental glasses showed dramatic changes after immersion in Tris buffer solution for periods of 1, 3, 6, 9 and 24 h. These changes related to ion exchange between the glass powder and the Tris buffer solution forming a silica-gel surface layer-Si(OH)_4_ on the glass surface after breaking of the Si-O-Si bonds. This was followed by leaching of calcium, phosphate and fluoride from the glass into the solution to form apatite. These findings were in agreement with the mechanism of apatite formation proposed by Hench [29, 30]. Furthermore, the ability of experimental glasses to form apatite in 6 h, compared with 24 h for the commercially-available 45S5(Sylc™), after immersion in Tris buffer solution, were consistent with previous findings of Farooq et al. [28] and Mneimne et al. [13].

### Orthodontic adhesive removal

It has previously been reported that TC bur increased enamel roughness compared to composite burs [31], white stone [32], stainburster bur [33] and adhesive residue remover [34]. These studies compared results with atomic force microscopy, profilometry and 3D scanning in blue-light technology. In the present study, the profilometry data revealed that the use of the TC bur in removing residual orthodontic adhesive increased enamel surface roughness regardless of the type of adhesive used. 45S5(Sylc™)-air-abrasion also produced an increase in the enamel roughness to some extent. These findings are in agreement with Banerjee et al. [7], who demonstrated that removal of residual adhesive resin (Unite™) using alumina air-abrasion caused more enamel loss (0.0386 mm^3^), followed by the TC bur (0.285 mm^3^) and finally 45S5 air-abrasion (0.135 mm^3^). In the present study, the novel experimental glass (QMAT3) induced less enamel roughness compared with the TC bur and 45S5(Sylc™)-air-abrasion, irrespective of the adhesive material used, a finding which was corroborated using SEM imaging. This finding relates to the lower hardness value of QMAT3 which approximates but does not exceed that of the enamel surface. Therefore, propelling this glass powder was less likely to roughen the enamel surface, mitigating the associated risk of plaque accumulation and caries formation. Furthermore, the handling technique used in this study was similar to that used by Paolinelis et al. [16], who confirmed that using the aforementioned operating parameters increased the cutting efficiency of the air-abrasion technique. Consequently, using accepted clinical handling parameters it appears that this novel glass powder may be capable of selective removal of orthodontic adhesives without inducing deleterious abrasion of the enamel surface, although quantification of the volume of loss was not undertaken.

Further laboratory research in relation to the cutting efficiency of this approach is required prior to clinical application, although preliminary data suggests comparable levels of efficiency to other bioactive glass formulations. Adhesive removal took approximately half the time with the TC bur. This discrepancy might relate to the aggressive cutting associated with sharp cutting blades of TC bur while bioactive glass propulsion works by means of abrasion. Similar conclusions were reached by both Karan et al. [31] and Mohebi et al. [32], who reported that TC burs removed adhesive remnants faster than composite bur and white stone, respectively. Moreover, polishing with non-fluoridated pumice for 20 s has little effect on enamel surface roughness values. These findings mirror analogous studies involving use of non-fluoridated pumice for 10 to 30 s [35–37] suggesting that polishing does not affect either the grooves or pits induced by enamel clean-up methods.

The ability of QMAT3 glass to form apatite earlier than 45S5(Sylc™) offers further promise to induce enamel remineralization; this potential may lead to application in the management of both pit and fissure, and smooth surface enamel caries. Notwithstanding this, it is important to emphasize that the present research is limited by its ex vivo nature; as such, replication within an in vivo situation is required. Moreover, blinding of the investigator was not performed, risking the introduction of assessor bias. In addition, teeth were stored in deionized water rather than undergoing a simulated artificial aging process involving thermomechanical cycling, the latter approach may have been more clinically representative, although use of water storage remains an accepted approach [38]. However, in view of the promising physical and handling properties highlighted in the present study, the remineralization potential of QMAT3 for orthodontically-induced white spot lesions will be explored in ongoing laboratory research.

## Conclusions

A novel bioactive glass (QMAT3) with a lower hardness than 45S5(Sylc™) and enamel has been developed. Its bioactivity was proved by early apatite formation compared with a proprietary agent. QMAT3 was capable of selective removal of residual orthodontic adhesive without inducing enamel damage. It, therefore, shows promise as a viable alternative to adhesive removal with a TC bur.
